# Kin selection favors religious traditions: ancestor worship as a cultural descendant-leaving strategy

**DOI:** 10.1080/2153599X.2023.2215854

**Published:** 2023-06-05

**Authors:** Kerstin Stucky, Andy Gardner

**Affiliations:** School of Biology, University of St Andrews, St Andrews, UK

**Keywords:** Ancestor-descendant conflict, cooperation, cultural tradition, gene-culture conflict, inclusive fitness, kin selection, mathematical model, religion

## Abstract

Recent years have seen renewed interest in the role of religious systems as drivers of the evolution of cooperation in human societies. One suggestion is that a cultural tradition of ancestor worship might have evolved as a “descendant-leaving strategy” of ancestors by encouraging increased altruism particularly between distant kin. Specifically, Coe and others have suggested a mechanism of cultural transmission exploiting social learning biases, whereby ancestors have been able to establish parental manipulation of kin recognition and perceived relatedness as a traditional behavior, leading to increased altruism among co-descendants and thereby maximizing the ancestor’s long-term inclusive fitness. Here, we develop a demographically explicit model in order to quantify the resulting increase in altruism and concomitant “ancestor-descendant conflict”, and to determine the evolutionary feasibility of religiously motivated cultural norms that promote altruism among co-descendants. Our analysis reveals that such norms could indeed drive an overall increase in altruism with potential for ancestor-descendant conflict, particularly in low-dispersal settings. Moreover, we find that natural selection can favor traditions encouraging increased altruism towards co-descendants under a range of conditions, with the inclusive-fitness costs of enacting an inappropriately high level of altruism being offset by inclusive-fitness benefits derived from the cultural tradition facilitating kin recognition.

## Introduction

Humans regularly cooperate with distant or non-relatives, including unfamiliar individuals, on a scale that is exceptional within the animal kingdom (Melis & Semmann, 2010). This poses an evolutionary puzzle and researchers have attempted to solve this by drawing on a range of explanatory mechanisms such as direct, indirect or generalized reciprocity (e.g., Barta et al., 2011; Panchanathan & Boyd, 2004; Pfeiffer et al., 2005), the development and policing of social norms (e.g., Chudek & Henrich, 2011; Fehr & Gaechter, 2002; Fehr & Schurtenberger, 2018), and cultural group selection (e.g., Chudek & Henrich, 2011; Henrich, 2004; Richerson et al., 2016). In this context, it has been repeatedly suggested that religion might have functioned as a catalyst in the promotion of large-scale cooperation in humans (e.g., Atran & Henrich, 2010; Bulbulia, 2008; Bulbulia & Frean, 2010; Crespi, 2016; Crespi & Summers, 2014; Kiper & Sosis, 2014; Norenzayan et al., 2016; Powell & Clarke, 2012; Szocik, 2017; Wilson, 2002).

Some researchers (e.g., Clark & Coe, 2021; Coe et al., 2010; Coe & Palmer, 2008; Coe & Palmer, 2013; Palmer et al., 2008; Palmer et al., 2013; Palmer & Steadman, 1997; Steadman & Palmer, 2008) have taken a similar approach in their investigation of cultural traditions that specifically emphasize altruism among kin, both on a conceptual level and in their study of the ethnographic record. They propose that the introduction of traditions that are often found in religious systems, such as ancestor worship, has led to a significant increase in cooperative behavior among close and distant kin, and potentially among non-kin in the long run. For instance, Coe et al. (2010) suggest that individuals might have been manipulated to increase their altruism towards identifiable co-descendants of a common ancestor via the transmission of cultural norms promoting the cooperation among kin specifically, together with being given the means of recognizing said kin. By influencing their children such that they recognize and cooperate with distant kin as if they were close kin, and pass these teachings on to their own children, some ancestors might have encouraged cooperative behavior among their descendants to an extent beyond what would otherwise be predicted by kin selection, reciprocity, or cultural group selection. Under this view, religious traditions such as ancestor worship have thus ultimately served as a “descendant-leaving strategy” (Palmer & Steadman, 1997), i.e., to maximize the respective ancestor’s long-term inclusive fitness, with their genes having spread more successfully as a result.

Such norms might encourage behavior opposing an individual’s own inclusive fitness interests and hence give rise to what has been termed “ancestor-descendant conflict” (Coe et al., 2010), i.e., the extension of parent-offspring conflict (Trivers, 1974) to more distant ancestor-descendant relationships. Coe et al. (2010) present a model illustrating the proposed conflict and its resolution by calculating an expected amount of altruism within particular pairs of individuals of varying kinship from three quantities: the number of generations descended from a common ancestor, the degree of genetic relatedness between the respective co-descendants, and the success rate of parental manipulation. Importantly, the success rate of parental manipulation represents the strength of the ancestor’s influence on the degree of altruism expected between co-descendants in subsequent generations such that the resulting altruism might be greater than that corresponding to the individual’s assumed basic inclusive fitness interests, i.e., that which is expected from their genetic relatedness. Accordingly, the authors conclude that by considering the impact of parental manipulation as a traditional behavior, the increased altruism among distant kin in so-called traditional societies found in the ethnographic record can be explained.

This is an intriguing idea. However, Coe et al.’s (2010) model does not allow for the evaluation of the overall amount of altruism occurring in a population and hence the extent of ancestor-descendant conflict. In order to do that, one would need to know how frequently relatives of different degrees encounter each other. That is, in order to determine the amount of altruism expected from culturally taught norms and/or genetic relatedness, one would need to assess the probability of encounters between co-descendants in a group, which would be expected to vary with demographic circumstances, and which in turn would shape the extent and potential resolution of the proposed ancestor-descendant conflict. Moreover, it is difficult to see why a mechanism such as ancestor worship would not be counteracted by natural selection, e.g., by acting on the cognitive foundations which influence an individual’s susceptibility to supernatural concepts. In light of the proposed ancestor-descendant conflict, it is therefore reasonable to ask whether and when a cultural system such as ancestor worship could evolve.

We develop a demographically explicit model to quantify the overall amount of altruism in a population with a religious system of ancestor worship and the potentially ensuing ancestor-descendant conflict, exploring the discrepancy between the culturally intended altruism, i.e., the amount of altruism between co-descendants in a group expected from cultural norms, on the one hand, and the amount of altruism in a group expected from individuals acting according to their genetic relatedness, on the other, under a range of demographic settings. To assess the evolutionary feasibility of the suggested mechanism, we examine the inclusive fitness consequences for an actor who adopts a cultural norm – that promotes the identification of co-descendants and increased altruism towards them – either fully, partially, or not at all, under a range of success rates of parental manipulation and cultural norms. This enables us to derive comparative predictions about the conditions under which a cultural system such as ancestor worship could have evolved as well as some of its properties, that is, how the proposed ancestor-descendant conflict might have been resolved.

## Model

We closely follow Coe et al.’s (2010) model, which considers the uniparental transmission of a culturally taught trait promoting altruism in a lineage of female descendants. The authors’ calculations of genetic relatedness – with, for example, maternal sisters being related by one half – imply standard diploid autosomal inheritance, female monogamy, and outbreeding. The formula they use to calculate the expected amount of altruism further implies a proportional relationship between altruism and the relatedness valuation that individuals place upon their respective social partner. Coe et al. (2010) assume that this relatedness valuation is given by the individuals’ actual genetic relatedness plus a potential increase in the relatedness valuation due to the ancestral influence, with this increase being modulated by the success rate of parental manipulation. The level of altruism an individual exhibits if following the cultural rule is therefore proportional to the relatedness valuation encouraged by the cultural norm, which the authors assume to be unity, i.e., individuals are expected to value their co-descendants as they would value themselves. Importantly, the culturally transmitted trait as described by the authors includes both the means for the identification of co-descendants as well as the prescribed relatedness valuation determining the expression of altruism towards these.

We assume a large population divided into social groups, with each group containing *n* pairs of women and men raising children, and we focus on social interactions between these women. Mothers pass the cultural instructions on how to recognize co-descendants and a prescribed relatedness valuation *R* for these, i.e., the ‘cultural coefficient of relatedness,' on to their daughters. Upon reaching maturity, daughters either disperse with probability *d* or else remain in their natal groups with probability 1-*d*, and sons always disperse, with dispersers traveling sufficiently far to ensure that they do not encounter relatives in their new groups, which ensures outbreeding as assumed by Coe et al. (2010). Further, we assume non-overlapping generations, group fissioning, and density-dependent regulation which maintains a constant number of groups of constant size across generations (cf. Stucky & Gardner, 2022). In line with Coe et al.’s (2010) suggested success rate of parental manipulation, daughters can accept, reject (or, equivalently, be ignorant of) or partially accept the cultural norm, and accordingly vary in their choice of social partners and their expression of altruism towards these during adulthood.

## Results

### Altruism expected from cultural norms

If individuals fully adopt a cultural norm which causes them to recognize and value co-descendants according to a cultural relatedness coefficient *R*, then we expect an overall amount of altruism *A*_culture_ = *γ* × *R*, where *γ* is the probability that two randomly chosen adult female group members are co-descendants. The probability that two adult females in generation *t *+ 1 are co-descendants is given by:

(1) $$\gamma_{t+1}=\;(1-d{)}^{2}\left(\frac{1}{n}+\left(1-\frac{1}{n}\right)\gamma_{t}\right).$$

That is, neither of them has dispersed which occurs with probability (1-*d*)^2^ and: either they share the same mother with probability 1/*n*, in which case they are co-descendants; or they have different mothers with probability 1-(1/*n*), in which case they are co-descendants if their mothers (of generation *t*) were co-descendants which occurs with probability *γ_t_*. So, at equilibrium (*γ_t _*_+ 1_ = *γ_t_* = *γ*) we obtain:

(2) $$\gamma =\frac{{\left(1-d\right)}^{2}}{n-(n-1){\left(1-d\right)}^{2}}.$$

**Figure 1.**
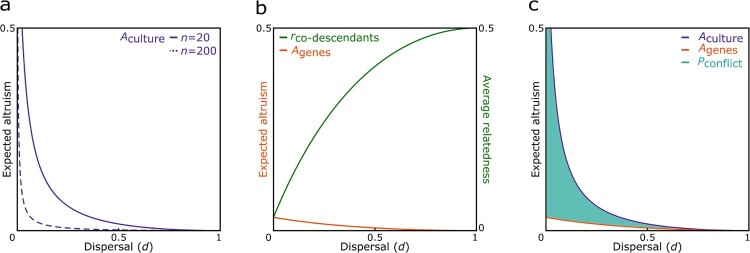
Ancestor worship can lead to ancestor-descendant conflict. **a)** In smaller groups (*n* = 20) ancestor worship can increase the overall level of altruism among co-descendants *A*_culture_ to a greater extent than in larger groups (*n *= 200) since average relatedness of co-descendants decreases with group size, **b)** the amount of altruism expected from genetic relatedness *A*_genes_ decreases with increasing dispersal whereas average relatedness between co-descendants *r*_co-descendants_ increases since it becomes more likely to encounter closely related opposed to distantly related co-descendants (here, *n* = 20), and **c)** the potential for ancestor-descendant conflict *P*_conflict_ is greatest in more viscous populations since the probability of meeting a co-descendant – which determines the culturally encouraged amount of altruism *A*_culture_ – would be high, but the average degree of genetic relatedness between co-descendants *r*_co-descendants_ – which modulates the genetically expected amount of altruism *A*_genes_ – would be low (here, *n* = 20).

Thus, the overall amount of altruism *A*_culture_ arising from full adoption of the cultural norm is:

(3) $$A_{\mathrm{culture}}=\frac{{\left(1-d\right)}^{2}}{n-(n-1){\left(1-d\right)}^{2}}\;R,\;$$

which is an increasing function of the cultural relatedness coefficient *R* and a decreasing function of both dispersal rate *d* and group size *n* (see Figure 1a). This means that a higher culturally prescribed relatedness valuation leads to a higher average amount of altruism *A*_culture_. More importantly, average altruism would be lower in populations containing larger groups with a higher dispersal rate than in more viscous populations containing smaller groups, since the likelihood of meeting a co-descendant is decreasing with increasing dispersal rate and group size.

### Altruism expected from genetic relatedness

If individuals behave according to their genetic relatedness, there are two scenarios to consider. On the one hand, if individuals cannot recognize their co-descendants in the absence of or due to fully rejecting a cultural norm promoting increased kin altruism, they will be expected to behave altruistically towards all group members according to the average genetic relatedness of group members in the population. On the other hand, if individuals partially accept the cultural norm such that they can use this information to enable them to recognize their co-descendants, but they reject the instructions to increase their altruism towards them, they will be expected to behave altruistically only towards their co-descendants and according to the average relatedness among them.

Consequently, if individuals cannot recognize their co-descendants and behave altruistically towards all group members according to their average genetic relatedness, we expect an overall amount of altruism *A*_genes_ = *r*_­­­group_, where *r*_group_ is the average genetic relatedness between group members, and is given by:

(4) $$r_{\mathrm{group}}=\;\gamma \;\times \;r_{\mathrm{co}-\mathrm{descendants}}+(1-\;\gamma )\;\times \;0,\;$$

i.e., a proportion *γ* of group members are co-descendants and are related on average by *r*_co-descendants_ and a proportion 1- *γ* are not co-descendants and are related by 0. The relatedness of co-descendants can be expressed as:

(5) $$r_{\mathrm{co}-\mathrm{descendants}}=\overset{\infty }{\underset{k=0}{\sum }}\delta_{k}r_{k},\;$$

where *δ_k_* is the probability that a co-descendant is a *k*th cousin, i.e., they are *k* generations descended from a common ancestor, and *r_k_* denotes the relatedness between *k*th cousins. Note that *δ_k_* = (1-*δ*_0_)*^k^ δ*_0_, where *δ*_0_ denotes the probability of a co-descendant being a sister and is given by *δ*_0_ = ((1-*d*)^2^/*n*)/*γ*, and that *r_k_* = (1/4)*^k^* 1/2. Making these substitutions obtains:

(6) $$r_{\mathrm{co}-\mathrm{descendants}}=\frac{2(n-(n-1){\left(1-d\right)}^{2})}{4n-(n-1){\left(1-d\right)}^{2}}.$$

Substituting eq. (6) into eq. (4) obtains:

(7) $$r_{\mathrm{group}}=\;\frac{2{\left(1-d\right)}^{2}}{4n-(n-1){\left(1-d\right)}^{2}}.$$

Therefore, the overall amount of altruism *A*_genes_ is:

(8) $$A_{\mathrm{genes}}=\frac{2{\left(1-d\right)}^{2}}{4n-(n-1){\left(1-d\right)}^{2}}.$$

If individuals are able to recognize their co-descendants and behave altruistically towards them according to their genetic relatedness, we expect an overall amount of altruism of *A*_genes_ = *γ* × *r*_co-descendants_ – and this level of altruism is exactly the same as that which arises when individuals are not able to recognize their kin, as derived above. That is, although kin recognition leads to co-descendants individually receiving more altruism, this increase is exactly offset by the reduction in the level of altruism received by non-co-descendants. This owes to Coe et al.’s (2010) assumption that expressed altruism is a linear function of relatedness valuation (cf. Faria & Gardner, 2020).

In either case, then, the level of altruism *A*_genes_ is a decreasing function of both dispersal rate *d* and group size *n* (see Figure 1b). Therefore, the average amount of altruism expected from genetic relatedness *A*_genes_ in a group would also be lower in populations with a higher dispersal rate and containing larger groups than in more viscous populations containing smaller groups, since the likelihood of meeting a co-descendant *γ* is reduced. This is despite the fact that average relatedness between co-descendants *r*_co-descendants_ would be higher in populations with a higher dispersal rate and larger groups, since it becomes more likely to meet a close – as opposed to a distantly related – co-descendant with increasing dispersal *d* and group size *n*, when meeting a co-descendant.

### Ancestor-descendant conflict

We find that the culturally encouraged and the genetically expected levels of altruism in a group can differ in the degree of overall amount of expressed altruism, i.e., there is potential for an ancestor-descendant conflict as anticipated by Coe et al. (2010), which is given by:

(9) $$P_{\mathrm{conflict}}=A_{\mathrm{culture}}-A_{\mathrm{genes}}=(1-d{)}^{2}\left(\frac{R}{1+(n-1)(1-{\left(1-d\right)}^{2})}-\frac{2}{3n+1+(n-1)(1-{\left(1-d\right)}^{2})}\right),\;$$

and which, as expected, is also a decreasing function of both dispersal rate and group size (see Figure 1c). Ancestors introducing a cultural tradition to increase altruism among their co-descendants would be expected to prescribe a cultural relatedness coefficient *R* equal to 1, so as to maximize their own inclusive fitness. Considering this, we find that the ancestor-descendant conflict would be greatest in populations containing larger groups with a low dispersal rate, since the probability of meeting a co-descendant – which determines the culturally encouraged amount of altruism *A*_culture_ – would be high, but the average degree of genetic relatedness between co-descendants – which modulates the genetically expected amount of altruism *A*_genes_ – would be low, whereas the conflict would be smallest in populations containing larger groups with a high dispersal rate, since both the probability of meeting a co-descendant and the average degree of genetic relatedness between co-descendants would be low.

### The evolutionary potential of ancestor worship as a descendant-leaving strategy

In order to assess the evolutionary feasibility of a cultural system such as ancestor worship, we investigate the inclusive fitness consequences for individuals in varying ecological scenarios and according to (i) whether they fully accept the cultural norm conveyed through ancestor worship, thus recognizing their co-descendants and treating them according to the cultural relatedness coefficient; or (ii) whether they fully reject the cultural norm (or, equivalently, are ignorant of it and of their kin relations altogether), thus treating everyone in the group according to the average genetic relatedness of group mates; or (iii) whether they only partially accept the cultural norm, thus recognizing their co-descendants and treating them according to a modulated cultural relatedness coefficient, i.e., exploring the effects of varying success rates of parental manipulation.

To do this, we need to specify an explicit inclusive fitness function. A simple functional form that complies with Coe et al.’s (2010) assumption that the amount of expressed altruism is proportional to the relatedness valuation the individual places upon her social partners is:

(10) $$W_{i}=\;\varepsilon -s\;1/2\;x^{2}+s\;x\;\rho ,\;$$

where: *ϵ* is an individual’s baseline fitness; *s* is her expected number of social partners; *x* is the amount of altruism she exhibits; and *ρ* is her genetic relatedness to her social partners. That is, the optimal level of altruism (i.e., satisfying d*W_i_*/d*x*_|*x*__= x* _= 0) is x* =* ρ*.

**Figure 2.**
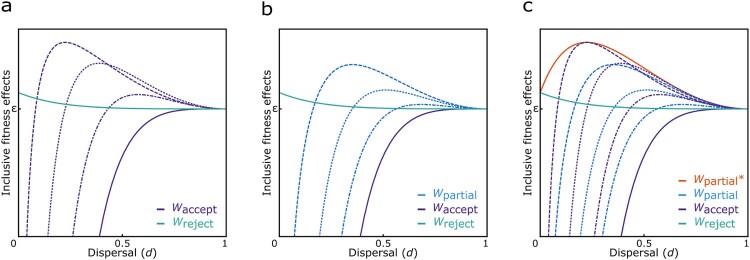
Inclusive fitness effects of ancestor worship. **a)** Fully accepting a cultural norm such as ancestor worship promoting increased altruism among co-descendants according to a cultural relatedness coefficient *R* (here, from left to right: *R* = ¼, ½, ¾, and 1) can increase an individual’s inclusive fitness (*W*_accept_) above that of an individual rejecting/being ignorant of such cultural norms (*W*_reject_) in a range of dispersal conditions (unless *R* = 1 or *d* = 1); **b)** accepting the cultural norm partially (here, from left to right: *χ* = ¼, ½, ¾) can increase an individual’s inclusive fitness (*W*_partial_) above that of an individual fully accepting the norm (*W*_accept_; for *R* = 1) and that of an individual rejecting/being ignorant of the cultural norm (*W*_reject_) in a range of dispersal conditions; and **c)** partially accepting the cultural norm such that an individual can recognize their co-descendants but treats them according to their genetic relatedness (for *χ* = 0) can increase the individual’s inclusive fitness (*W*_partial_*) above that of any of the other strategies in all conditions, unless everyone disperses. In all panels we assume *n* = 20.

If (i) an individual fully accepts the cultural norm, i.e., she recognizes her co-descendants and directs her culturally encouraged altruism towards them, then *s* = *γ *× (*n*-1), *x* = *R*, and *ρ* = *r*_co-descendants_. This means that a proportion *γ* of the *n*-1 other women in the group are identifiable co-descendants, who the focal individual treats as being valued by *R* according to the cultural relatedness coefficient, but who are genetically related to the focal individual by *r*_co-descendants_. For a cultural norm that encourages individuals to value their co-descendants as they would value themselves (i.e., *R* = 1), inclusive fitness is an increasing function of dispersal rate and a decreasing function of group size. Fully following this norm would, however, decrease an individual’s inclusive fitness relative to her baseline fitness in all ecological scenarios (i.e., for all 0 < *d *< 1 and *n* > 1), since she would be committed to express a level of altruism always exceeding that which would be expected from genetic relatedness. This effect becomes smaller with increasing dispersal, though, since the likelihood of encountering co-descendants decreases whereas the average relatedness of co-descendants increases. Allowing for variation in the cultural relatedness coefficient (i.e., for *R* < 1), however, leads to a range of scenarios in which an individual’s inclusive fitness could be increased relative to her baseline fitness (see below and see Figure 2a).

If instead (ii) an individual rejects the cultural norm altogether or if there is no such norm in place, i.e., she does not recognize her co-descendants and treats everyone in her group according to the group’s average genetic relatedness, then *s* = *n*-1, *x* = *r*_group_, and *ρ* = *r*_group_. This behavior would also increase an individual’s inclusive fitness relative to her baseline fitness. This effect, however, is a decreasing function of increasing dispersal and group size (for all 0 < *d *< 1 and *n* > 2; see Figure 2a). Alternatively, if (iii), in line with Coe et al.’s (2010) suggested success rate of parental manipulation, individuals only partially accept the culturally encouraged relatedness valuation, then *s* = *γ *× (*n*-1), *x* = *θ*, and *ρ* = *r*_co-descendants_, where *θ* = *χ* × 1 + (1- *χ*) ×* r*_co-descendants_ is the relatedness valuation resulting from parental manipulation by an extent *χ*. That is, the relatedness valuation is a weighted average of the culturally encouraged value of 1 and the actual genetic relatedness *r*_co-descendants_, with *χ* providing the relative weight placed on the cultural value. Following the cultural norm only partially (i.e., for all *χ *< 1) would also increase an individual’s inclusive fitness relative to her baseline fitness in a range of scenarios (see Figure 2b). This effect, however, is a decreasing function of the success rate of parental manipulation, since the individual would increase her relatedness valuation of co-descendants beyond what would be appropriate from her gene’s perspective (i.e., for all *χ *> 0). This becomes clear in the specific case of an individual who partially accepts the norm such that she recognizes her co-descendants and directs her altruism towards these but rejects the culturally prescribed relatedness valuation (i.e., for *χ *= 0). Here, inclusive fitness is an increasing function of dispersal rate and group size at lower dispersal rates and a decreasing function of dispersal rate and group size at higher dispersal rates, eventually approaching zero when approaching full dispersal. Importantly, the inclusive fitness effects of this strategy are exceeding the effects of any other behavior in any of the conditions – all else being equal – since the individual would be able to recognize and direct her altruism towards her co-descendants in a way that is optimal from her gene’s perspective (see Figure 2c).

Comparing the inclusive fitness effects for individuals performing the aforementioned strategies, we find that the inclusive fitness of individuals following a cultural norm that encourages them to recognize and direct increased altruism towards co-descendants according to a cultural coefficient of relatedness fully or partially (for *χ* > 0) would always be lower than the inclusive fitness of individuals only partially accepting the norm such that they recognize co-descendants but treat them according to their genetic relatedness (i.e., for *χ* = 0, unless *R* = *r*_co-descendants_), all else being equal. A cultural system solely involving kin recognition would therefore be more beneficial than a system involving kin recognition and increased kin altruism. Consequently, we find that natural selection would not favor a system such as ancestor worship as a cultural promoter of increased kin altruism to evolve in a population if kin recognition was uncoupled from the norm encouraging the increase in altruism, under the assumptions of our model. However, if the identification of co-descendants was promoted via cultural traditions related to ancestor worship as proposed by Coe et al. (2010), such that individuals brought up in a system without or rejecting these traditions could not recognize their co-descendants, we find that natural selection could favor cultural traditions which promote altruism between co-descendants exceeding that which is expected from genetic relatedness in a range of conditions, i.e., when the inclusive fitness of an individual following the cultural norm exceeded that of an individual ignorant of the traditions and thus their kin relations.

### When does natural selection favor ancestor worship?

In order to determine when ancestor worship would be favored by natural selection we determine the conditions under which adoption of ancestor worship improves the individual’s inclusive fitness relative to what it would be if the individual fully rejected or was fully ignorant of the cultural tradition. This is where the benefits that come from being able to identify co-descendants outweigh the costs of enacting an inordinate amount of altruism towards them. Specifically, we identify the value of *R* which represents the maximum cultural relatedness coefficient and likewise, the value of *χ* which represents the maximum success rate of parental manipulation such that the individual breaks even in terms of these benefits and costs balancing out. We expect that ancestors would want to drive the accepted cultural relatedness coefficient as high as possible in order to maximize their inclusive fitness. Accordingly, the maximum cultural relatedness coefficient (i.e., satisfying *γ* × (*n*-1) (-½ *R*^2^+ *R r*_co-descendants_) = (*n*-1) (-½ *r*_group_^2^ + *r*_group_^2^)) is:

(11) $$R_{\max}=\frac{2(1+(n-1)(1-{\left(1-d\right)}^{2})+\sqrt{n(1+(n-1)(1-{\left(1-d\right)}^{2}))(1-{\left(1-d\right)}^{2})}}{3n+1+(n-1)(1-{\left(1-d\right)}^{2})},\;$$

and the maximum success rate of parental manipulation (i.e., satisfying *γ* × (*n*-1) (-½ (*χ* × 1 + (1- *χ*) × *r*_co-descendants_)^2^ + (*χ* × 1 + (1- *χ*) × *r*_co-descendants_) *r*_co-descendants_) = (*n*-1) (-½ *r*_group_^2^+ *r*_group_^2^) for *χ *> 0) is:

(12) $$\chi_{\max}=\frac{2\sqrt{n(1+(n-1)(1-{\left(1-d\right)}^{2}))(1-{\left(1-d\right)}^{2})}}{3n-n(1-{\left(1-d\right)}^{2})-{\left(1-d\right)}^{2}}.$$

**Figure 3.**
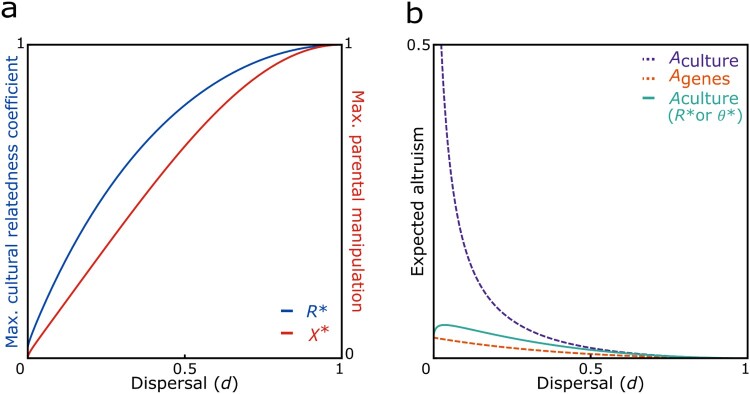
Potential ancestor-descendant conflict resolution. **a)** The introduction of norms leading to an intermediate degree of altruism between co-descendants via an ecologically variable cultural relatedness coefficient R* or success rate of parental manipulation χ*, respectively, allows for the evolution of ancestor worship in a range of conditions; and **b)** can lead to a reduction of the discrepancy between the culturally promoted amount of altruism *A*_culture_ and the amount of altruism expected from genetic relatedness *A*_genes_, indicating a resolution to the proposed ancestor-descendant conflict. In both panels we assume *n* = 20.

Replacing *χ* with *χ*_max_ in the expression for *θ*, we recover the expression for *R*_max_; i.e., effectively the potential maximum amount of expressed altruism among co-descendants due to ancestral influence on their perceived relatedness, which is evolutionarily feasible from the perspective of the manipulated individual’s inclusive fitness. Reasonable approximations for these quantities are obtained by expressing them in the limit of infinite group size, with $R_{\max}\;=\;\theta_{\max}\;=4(1-{\left(1-d\right)}^{2})/(4-{\left(1-d\right)}^{2})$ and $\chi_{\max}\;=2(1-{\left(1-d\right)}^{2})/(3-(1-{\left(1-d\right)}^{2}))$. These provide good approximations for even relatively small values of *n* (i.e., for *R*_max_ and *θ*_max_: within 5% of error for *n *> 56, for all *d *≥ 0.1; and for *χ*_max_: within 5% of error for *n* > 46 for all *d*). These approximations of *R*_max_, *θ*_max_ and *χ*_max_ are all increasing functions of dispersal rate. Numerical investigation suggests that this is also the case even for smaller values of *n*, where the approximations do not hold as closely. Following from this, we find that in populations with a higher dispersal rate it is more likely for cultural traditions promoting altruism towards co-descendants to evolve and/or be sustained at a comparatively higher degree in large groups (see Figure 3a). More importantly, the introduction of a cultural coefficient of relatedness *R*_max_ and a success rate of parental manipulation *χ*_max_ as functions of dispersal rate and group size leads to a range of potential cultural traditions resulting in an intermediate degree of altruism between co-descendants and a significant reduction of the discrepancy between the amount of altruism intended by ancestors and the amount of altruism expected from genetic relatedness, providing a resolution to the proposed ancestor-descendant conflict (see Figure 3b).

## Discussion

It has been repeatedly suggested that religious beliefs and behaviors have played an important role in facilitating the unusual extent of cooperation found in human societies. For instance, it has been proposed that the introduction of cultural traditions such as ancestor worship as a descendant-leaving strategy has led to increased altruism specifically between distant kin. More precisely, Coe et al. (2010) have presented a mechanism of cultural transmission exploiting social learning biases, by which ancestors might have been able to establish parental manipulation of kin recognition and perceived relatedness as a traditional behavior, eventually leading to increased altruism among co-descendants and thereby maximizing the respective ancestor’s inclusive fitness. Here, we developed a demographically explicit model to quantify the proposed increase in altruism, assess the associated potential for ancestor-descendant conflict, and investigate the evolutionary feasibility of religiously motivated cultural norms promoting increased altruism among co-descendants. Our analysis reveals that such norms could indeed generate an overall increase in altruism with potential for ancestor-descendant conflict as Coe et al. (2010) had anticipated. Moreover, we find that kin selection could favor cultural traditions promoting increased altruism among co-descendants under a range of conditions.

More specifically, our demographically explicit model allows us to take Coe et al.’s (2010) ideas of cultural norms encouraging increased altruism among kin and investigate their consequences in terms of overall levels of altruism in the population. Given that individuals accept these norms, we find that they could lead to a strong increase in overall altruism, specifically in more viscous populations made up of smaller groups as compared with populations characterized by a higher dispersal rate and larger groups, since it becomes less likely to meet a co-descendant with increasing dispersal and group size. Accordingly, we find potential for ancestor-descendant conflict as anticipated by the authors, and in addition, our analysis reveals that the extent of this conflict would be greatest in large groups in more viscous populations. More importantly, we find that natural selection could favor traditions encouraging increased altruism towards co-descendants in a range of conditions, given a demographically variable rate of ancestral manipulation and given that information about kin relations is strongly tied to the respective norms promoting increased altruism among kin. This would allow individuals to direct their altruistic behavior towards co-descendants as opposed to non-kin, thereby offsetting some of the inclusive fitness costs incurred by the increase in expressed altruism. The inclusive fitness benefits due to kin recognition will vary, however, depending on the specific demography and hence relatedness structure of a population. For instance, in more viscous populations it would be less costly for individuals to behave indiscriminately altruistic, since they would be more likely to be surrounded by relatives, and kin recognition would therefore have less of an impact. Accordingly, ancestors might be expected to attain a higher rate of manipulation of their descendants’ perceived relatedness in populations of large groups and a high rate of dispersal as opposed to more viscous populations. In these cases, we might expect increased altruism towards distant kin to occur, since here both ancestors and descendants would be able to maximize their inclusive fitness, providing a resolution to the proposed ancestor-descendant conflict.

Previously, it has been suggested that religious cognition and behavior might have originated as a product of kin selection, and more specifically as a means to suppress intra-family conflict (Crespi, 2016; Crespi & Summers, 2014). Indeed, kin selection could favor religiosity (Stucky & Gardner, 2022), i.e., the susceptibility to supernatural concepts, such that individuals may be manipulated into cooperative behavior towards related social partners by using culturally evolved narratives about supernatural entities. A culturally transmitted trait containing such narratives and exploiting this susceptibility as well as social learning biases could indeed be represented by the religious practice of ancestor worship, i.e., “the communicated acceptance of the claim that dead ancestors influence and/or are influenced by their living descendants” (Clark & Coe, 2021). The veneration of specific deceased kin – genealogical, cultural, or mythical – has been suggested to be a widespread and diverse phenomenon in past and present societies and is regarded to play an important role for social cohesion and organization (e.g., see Couderc & Sillander, 2012, for a summary of the ethnographic literature and conceptions of ancestor worship/ “ancestorship” in general and an in-depth overview of ancestor worship in Borneo societies; see Steadman et al., 1996, for a view on the universality of ancestor worship; and see Peoples et al., 2016, for an opposing view on the distribution and phylogenetic history of ancestor worship).

For example, evidence from colonial accounts and the archaeological record point to elaborate, long-lived, and widespread practices of ancestor cult in prehistoric Andean societies (Hastorf, 2003; Lau, 2021; Mantha, 2009). Local kin groups (*ayllu*) regularly interacted with their respective founding ancestors (*mallqui*) in rituals involving the ancestors’ mummified bodies and other cult objects representing the venerated deceased, such as stone effigies. These devotional practices for individuals, who were perceived as valued family members, and the collective effort put into the production of their effigies are suggested to have promoted the descendants’ group identity (Lau, 2021). By the time of the arrival of the Spanish colonialists, some of these groups had developed into complex, more inclusive, social units consisting of several kin collectives. These collectives were organized hierarchically according to the relative genealogical distance of their respective ancestors to the founding progenitor of the larger community, with the associated above-ground mortuary structures representing the territorial and social boundaries of different groups (Mantha, 2009). Moreover, archaeological evidence from the Titicaca Basin indicates that, following the decline of the Chiripa culture (around 250 BC), the focus of ancestor veneration shifted from the female to the male in this region (Hastorf, 2003).

Among present-day Bentian communities in southeast Borneo, ancestors are often invoked in rituals and public discourse as sources of potency, authority, and morality. They can take various forms and be addressed individually or as an anonymous collectivity and take on different, context-dependent roles. Importantly, in their collective role of “elders who came before” (*ulun tuha one*) they represent the moral ideals of customary law (*adat*), thereby promoting “socio-centric values which encourage integration and relation affirming behavior” (Sillander, 2012). Individually invoked ancestors as genealogical forebears of status and/or resources can function to integrate as well as differentiate groups, however. Furthermore, some revered ancestors have attained their status due to their importance to the community, with no actual genealogical links to their devotees (Sillander, 2012), reflecting the rather flexible and inclusive bilateral kinship system of Bentian groups (Sillander, 2016). In contemporary China, ancestor veneration remains to be an important cultural tradition in many provinces, too, despite major political and demographic shifts of the last 100 years. Next to regular visits to the gravesites of ancestors, families are obliged to maintain a family genealogy which familiarizes members with the structure of the kinship system, their position therein, as well as their responsibilities. One central responsibility of the descendants concerns the continuation of the male family line. And indeed, a recent study investigating the demographic implications of ancestor worship in China found positive correlations of involvement in ancestor worship practices with lower age at marriage, more offspring, a higher probability of having at least one son, and more sons in general (Hu & Tian, 2018).

Here, we have investigated how ancestor worship might have evolved as a descendant-leaving strategy as proposed by Coe et al. (2010), and consequently influenced the extent and direction of cooperative behavior in human societies. To do this, we have adopted the authors’ assumptions of proportionality of expressed altruism and relatedness, uniparental transmission of the cultural trait in a lineage of female descendants, female monogamy, and outbreeding, in the design of our model. However, this constrains our analysis such that it ignores the possibility of altruism being a non-linear function of relatedness as well as more realistic scenarios allowing for variation in general demography and individual costs and benefits. For instance, owing to the assumption of proportionality of expressed altruism and relatedness, the increase in overall altruism predicted by our analysis is entirely based on the increased relatedness valuation promoted by ancestor worship. Yet, if we assume that altruism is a convex function of relatedness, the promotion of kin recognition could lead to an additional increase in the overall amount of altruism, whereas assuming that altruism is a concave function of relatedness, the promotion of kin recognition could lead to a decrease in overall amount of altruism (cf. Faria & Gardner, 2020). In either case, we would expect this to alter the extent of the potential for ancestor-descendant conflict.

Furthermore, our model investigates a sex-specific cultural trait assuming a strongly sex-biased demography in favor of the respective sex. Allowing for a less sex-biased demography or a demography biased towards the other sex would possibly alter the relatedness structure of a population and the associated trade-offs for individuals and might therefore result in different conditions under which ancestor worship could evolve. For instance, mating systems causing reproductive skew or sex differences in dispersal rate can potentially lead to sex differences in levels of religiosity owing to the resulting sex differences in relatedness between group members (Stucky & Gardner, 2022). It could be expected that such individual variation in the susceptibility to supernatural concepts have had an impact on the cultural transmission of a trait exploiting this susceptibility, aside from potential sex differences in individual costs and benefits that could arise from sexual selection (e.g., see Andersson, 1994). In addition, we have assumed group fissioning such that there are no kin competition effects under limited dispersal (Gardner & West, 2006). Allowing for other dispersal scenarios could lead to the increase of local resource competition among kin and might therefore affect our results relating to the maximum rate of ancestral manipulation. In the future, it could therefore be interesting to explore how variation in demographic factors and individual costs and benefits might influence the conditions for a cultural trait such as ancestor worship to evolve, and ultimately to align our model’s assumptions with more complex real-world examples as described above in order to test our predictions.

Nevertheless, from our analysis we can see how a cultural system exploiting cognitive biases to promote increased altruism among kin could generally arise, given the counterbalancing inclusive fitness effects of kin recognition. Ancestors who introduced cultural traditions such as ancestor worship might therefore have been more successful in leaving descendants as has been proposed, potentially resulting in the spread of such cultural traditions. And since groups with these traditions might have been more altruistic overall than groups lacking these, it would be useful to investigate the effects of such traditions at the between-group level, specifically including scenarios where the cultural manipulation of perceived relatedness might have been extended to non-kin, i.e., in networks of “fictive kinship” (Calhoun, 2002). In conclusion, cultural traditions such as ancestor worship might have been favored by kin selection with potential implications for the cultural evolution of religious systems.
